# Biogenetic Relationships of Bioactive Sponge Merotriterpenoids

**DOI:** 10.3390/md15090285

**Published:** 2017-09-10

**Authors:** Thomas E. Smith

**Affiliations:** 1 Department of Medicinal Chemistry, University of Utah, 30 S. 2000 E., Salt Lake City, UT 84112, USA; 2 Department of Integrative Biology, University of Texas at Austin, 2506 Speedway, NMS 4.216 stop C0930, Austin, TX 78712, USA

**Keywords:** sponge, meroterpenoid, marine natural product, medicinal chemistry, biosynthesis, drug discovery

## Abstract

Hydroquinone meroterpenoids, especially those derived from marine sponges, display a wide range of biological activities. However, use of these compounds is limited by their inaccessibility; there is no sustainable supply of these compounds. Furthermore, our knowledge of their metabolic origin remains completely unstudied. In this review, an in depth structural analysis of sponge merotriterpenoids, including the adociasulfate family of kinesin motor protein inhibitors, provides insight into their biosynthesis. Several key structural features provide clues to the relationships between compounds. All adociasulfates appear to be derived from only four different hydroquinone hexaprenyl diphosphate precursors, each varying in the number and position of epoxidations. Proton-initiated cyclization of these precursors can lead to all carbon skeletons observed amongst sponge merotriterpenoids. Consideration of the enzymes involved in the proposed biosynthetic route suggests a bacterial source, and a hypothetical gene cluster was constructed that may facilitate discovery of the authentic pathway from the sponge metagenome. A similar rationale can be extended to other sponge meroterpenoids, for which no biosynthetic pathways have yet been identified.

## 1. Introduction

**Figure 1:**
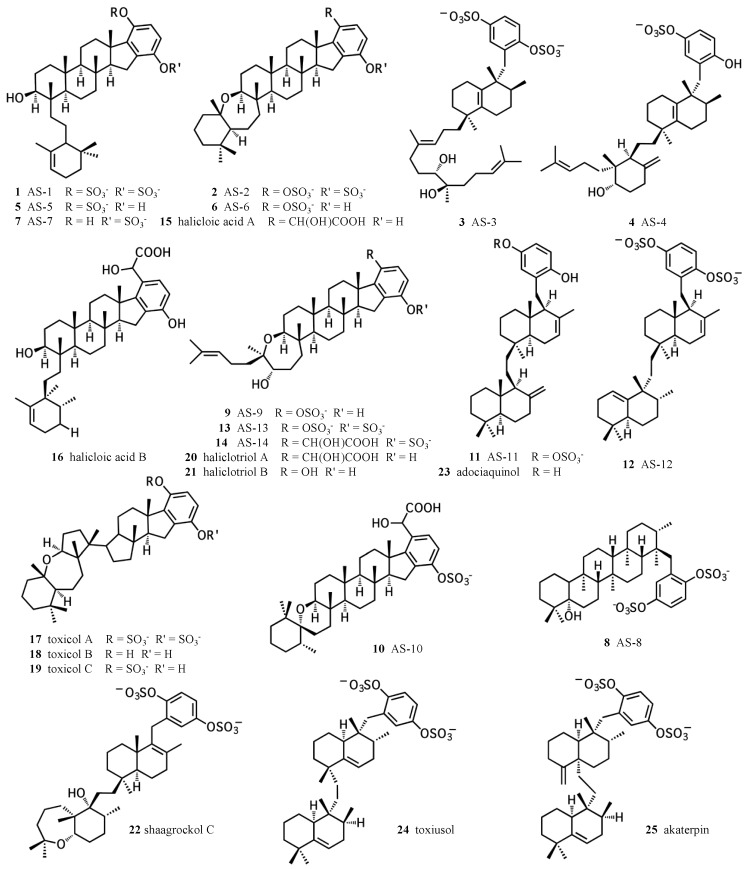
Chemical structures of sponge hydroquinone merotriterpenoids. The adociasulfates and related compounds are derived from sponges of the family *Chalinidae*, with the exception of akaterpin, isolated from *Callyspongia sp*.

Meroterpenes have long been recognized for their diverse biological activities. In particular, hydroquinone meroterpenes are interesting because of their potential for redox chemistry and wide distribution in nature [1,2]. Marine sponges represent a prolific source of hydroquinone meroterpenoids, some of which exhibit unique activities that cannot be substituted for using alternative compounds. The diversity of structures and activities of sponge hydroquinone meroterpenoids have been thoroughly reviewed by Menna et al. [1]. This review focuses on the merotriterpenoids, including the adociasulfates (Figure 1). This family includes several unique carbon skeletons, its members are frequently sulfated, and it also encompasses a wide variety of biological activities. The toxicols (**17**–**19**) and shaagrockol C (**22**) inhibit the DNA polymerase function of HIV-1 reverse transcriptase [3]. Akaterpin (**25**) inhibits hydrolysis of phosphatidylinositol by phospholipase C, a key step in eukaryotic signaling pathways by its production of diacylglycerol and inositol triphosphate [4]. Indoleamine 2,3-dioxygenase, whose activity mediates T-cell activation and whose overexpression in cancer may prevent tumor rejection, is inhibited by halicloic acids A and B (**15**, **16**) [5]. Some of these compounds also display weak antimicrobial activities [6,7]. The adociasulfate family has been shown to inhibit H^+^-ATPases and kinesin motor proteins [8,9,10,11]. Inhibition of kinesin by adociasulfate-2 (**2**) involves competition with microtubules for binding [11,12]. This mode of kinesin inhibition is known for only two other compounds, rose bengal lactone and the polyoxometalate NSC 622124, which both display characteristic features of nonspecific inhibition, including aggregate formation, indiscriminate binding to positively charged protein surfaces, and inhibition of a variety of enzyme activities [13,14,15,16,17]. Thus, sponge hydroquinone merotriterpenoids display a variety of therapeutically interesting activities, including some with unique mechanisms of action.

The adociasulfates in particular, with their unique mechanism of action, are not only interesting from a medicinal perspective, but have great potential as tools for studying the function of kinesins in cell biology. Neurons rely on precise intracellular organization and transport to function, as different cellular regions have very distinct roles in responding to and relaying signals. Use of **2** revealed a role for kinesin motor proteins in the transport of cytoskeletal filaments within axons [18], and in intracellular spatio-temporal control of gene expression via transport of synapse-specific mRNAs [19]. Kinesins are also involved in reconstructing the nucleus after cell division. Formation of nuclear pore complexes (NPCs) in *Xenopus laevis* eggs was inhibited by **2**, but not the double membrane of the nuclear envelope (NE), indicating the existence of a distinct vesicle population for delivering NPCs that utilize kinesin-guided microtubule transport [20]. Developmental processes have also been probed using **2**. Asymmetric, kinesin-dependent shuttling of cargo was shown to occur very early in the development of frog and chick embryos, suggesting a cytoskeletal role in establishing left-right asymmetry [21]. Treatment of early embryos with **2** led to disruption of this asymmetry. Finally, adociasulfates have been used to interrogate kinesin function directly. The kinesin microtubule binding site was mapped based on binding experiments with **2** [22], and, more recently, adociasulfates were shown to display affinity for non-kinesin microtubule binding sites, indicating their potential as probes of other microtubule-binding proteins [12]. 

**Figure 2:**
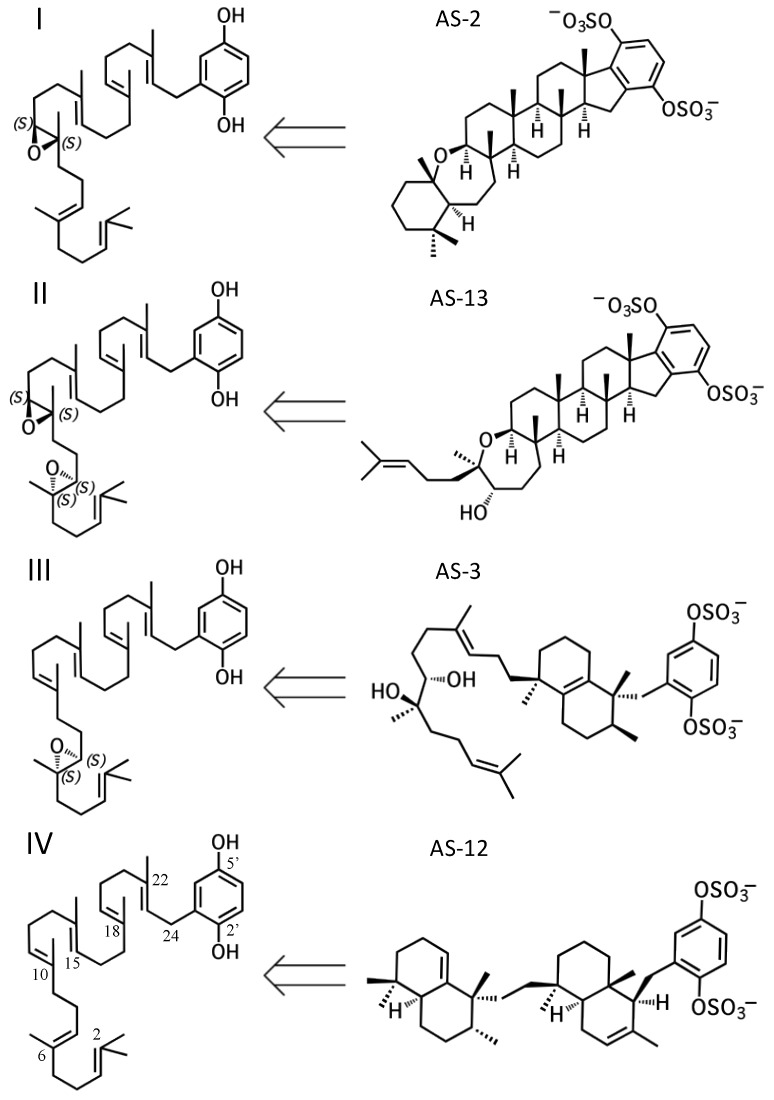
The four, putative hydroquinone merotriterpenoid biosynthetic classes. Sponge merotriterpenoids can be divided into four groups by the number and position of epoxidations of the linear hexaprenoid precursor (left side). Representative adociasulfates of each major group are shown (right side).

Despite their useful biological activities, sponge meroterpenoids are often unobtainable due to a lack of practical chemical syntheses and the difficulties associated with obtaining material from biological sources [23,24,25,26]. For this reason, studies using these compounds in biological applications are scarce and relatively infrequent. For example, with the exception of the most recent study, all of the studies described above obtained **2** from the authors of its original publication [11]. Thus, there is a need for a sustainable means of producing such compounds in order to make full use of their potential. This could be accomplished using a biosynthetic approach. However, there is a lack of knowledge with regard to meroterpenoid biosynthesis in marine invertebrates. No pathways for such compounds have been described despite hundreds of known compounds [1]. The characterization of one meroterpenoid pathway could reveal other the existence of other pathways, as sponge-derived hydroquinone meroterpenoids share many overlapping structural features that suggest common metabolic origins. To this end, adociasulfates provide an excellent starting point because a relatively simple biosynthetic hypothesis can be derived from a limited number of precursors (Figure 2). In fact, it is conceivable that all sponge triterpene hydroquinones are derived from a single parent pathway. The purpose of this review is to draw attention to the structural relationships between compounds and show that a thorough analysis of these relationships can reveal clues to their biosynthetic origin. Below, I discuss the features that unify the adociasulfates and other merotriterpenoids, make a case for the enzymes that are likely to be involved in their construction, and establish a biogenetic hypothesis. This analysis results in a hypothetical, bacteria-derived adociasulfate pathway. 

## 2. A proposed biosynthetic route for sponge hydroquinone merotriterpenoids

A defining feature of the adociasulfates is that the arrangement of methyl groups implies a linear triterpene-diphosphate precursor, as opposed to squalene. Prenyl diphosphates are typically formed by a head-to-tail condensation of isopentenyl diphosphate (IPP) with either dimethylallyl diphosphate (DMAPP) or the product of a previous such condensation, yielding linear terpenes extended by five carbons. Squalene, however, is made by the tail-to-tail condensation of two C_15_ farnesyl-diphosphates (FPP) to produce a symmetrical triterpene. The consequences of this are twofold. First, without the diphosphate, squalene is no longer activated for prenyl transfer to a hydrobenzoquinoid substrate. Second, cyclized derivatives of squalene display a characteristic arrangement of methyls that is not observed for adociasulfates or any other hydroquinone meroterpenoids. Linear meroterpenoids have been reported from sponges before, though not from sponges that produce adociasulfates [1]. Nonetheless, there is a precedent for prenyl transfer of linear triterpenes to quinones, resembling ubiquinone biosynthesis, while there is none for the equivalent transfer of squalene.

**Figure 3:**
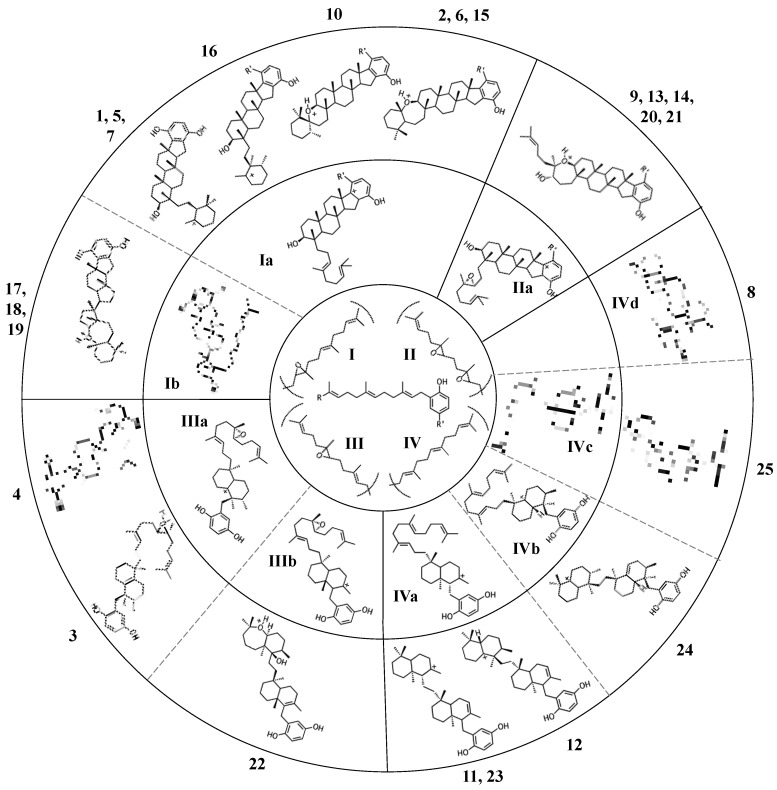
Diverse merotriterpenoid skeletons can be derived from a small number of related precursors via different cyclization routes. The innermost circle shows the four putative precursor molecules. Carbocation products of a single cyclization event are shown in the middle ring. Carbocation products of a second cyclization event are shown in the outermost ring. Numeric designations of final products lie outside of the circle. The number and complexity of structures expands outwards from the simple precursors in the center. The R group of the center linear precursor is substituted with one of the four groups in shown in parentheses, while the R’ group denotes a hydroxyl group for most compounds, or a glycolic acid moiety for some group I and II compounds.

All sponge merotriterpenoids can potentially be derived from a common series of linear precursors (Figure 2). These universal precursors, the products of aromatic prenylation by hexaprenyl diphosphate, would then be cyclized via a proton-initiated (type II), carbocation-mediated cyclization cascade. Most adociasulfates are hydroxylated at one (e.g., **1**, **2**) or two (e.g., **13**) carbons at positions corresponding to alkenes in hexaprenyl diphosphate. This suggests that epoxidation of the linear substrate occurs prior to cyclization. The number and position of epoxides in the cyclization substrate provides a convenient way to group biosynthetically related sponge merotriterpenoids. Thus, group I precursors are epoxidized at position 10,11, group II at both positions 6,7 and 10,11, and group III at position 6,7, while group IV compounds are not epoxidized. All proposed cyclization schemes described herein are based on (*S,S*) epoxide configurations, as predicted from the configurations of the putative epoxide-derived hydroxy carbons present in group I, II, and III adociasulfates. A variety of skeletons resulting from multiple cyclization events of these precursors is shown in Figure 3.

### 2.1. Group I compounds

**Figure 4:**
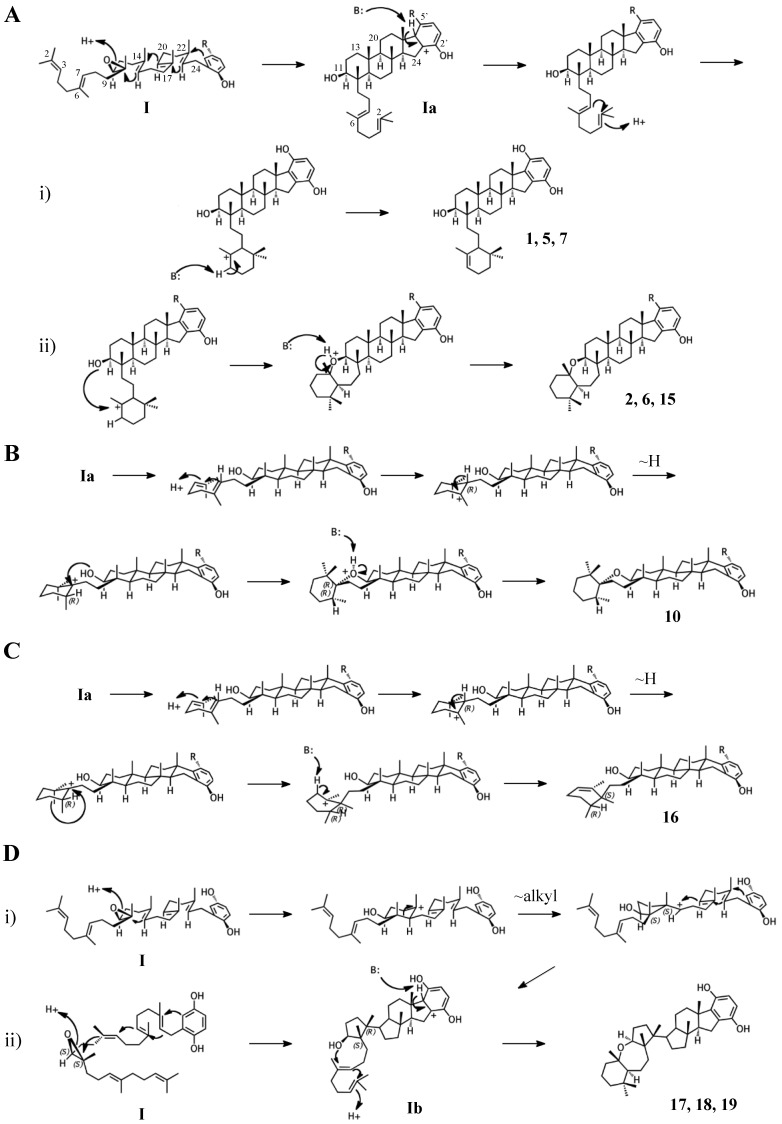
Putative cyclization routes of group I sponge merotriterpenoids, derived from 10,11-epoxyhexaprenyl diphosphate, including: (**A**) **1**, **2**, **5**, **6**, **7**, halicloic acid A (**15**), (**B**) **10**, (**C**) halicloic acid B (**16**), and (**D**) toxicols A-C (**17**–**19**).

The simplest hypothetical cyclization schemes involve the group I meroterpenoids. Compounds in this group likely undergo two independent cyclization cascades and exhibit few rearrangements. The initial cyclization of **1**, **2**, **5**, **6**, **7**, and halicloic acid A (**15**) would be identical for each compound, with epoxide opening to form a hydroxyl group at C11, establishing the sterol-like, four-ring system with ring D fused to the hydrobenzoquinone moiety (Figure 4A) [5,9,10]. The resulting carbocation would then be quenched by proton abstraction, restoring aromaticity. A second proton-initiated cyclization of the remaining two olefins would produce a fifth ring and a carbocation at position C6. Here, **1**, **5**, and **7** would differ from **2**, **6**, and **15** in the manner of base abstraction. In the former group, deprotonation would occur at C5 to introduce a new double bond, leaving the fifth ring independent of the core. In the latter group, a sixth, seven-membered ring would be formed by attack of the C11 hydroxyl on the C6 carbocation. Proton abstraction would then occur at the cyclic ether oxygen. AS-10 (**10**) could be obtained from the same initial cyclization, but would involve a hydride shift in the second cyclization event, placing the carbocation on C7 instead of C6 and resulting in a six-membered heterocycle (Figure 4B) [8]. The 3D structure of **2** would be flat relative to **10**, whose terminal ring would be twisted perpendicular to the plane of the core ring system. Halicloic acid B (**16**) resembles **10**, but the second cyclization event would involve an additional rearrangement: a methyl transfer following the hydride shift, placing the carbocation on C2 (Figure 4C) [5]. Deprotonation at C3 would then yield a tri-substituted alkene. A glycolic acid moiety substitutes for the 5′ hydroxyl in **10**, **15**, and **16**, suggesting an alternative aromatic prenyl acceptor to hydrobenzoquinone may be used. The final group I terpenes, toxicols A-C (**17**–**19**), likely undergo a unique cyclization that could occur in two different ways. In the first, an alkyl shift would condense the initial six-membered ring into a five-membered ring, resulting in an unstable secondary carbocation at C15 (Figure 4(Di)) [7]. Cyclization would then continue with subsequent attack on the C15 carbocation by C19. In the second, the initial epoxide opening would involve a direct attack by the 14,15-olefin on C10, which would be sterically hindered by the two methyls of C10 and C14 (Figure 4(Dii)). A second cyclization step and proton abstraction would result in the final product, with two independent ring systems. Finally, adociasulfates and related meroterpenoids would be sulfated at either, none, or both hydrobenzoquinone hydroxyls, while 5′ glycolic acids appear not to be modified further.

### 2.2. Group II compounds

**Figure 5:**
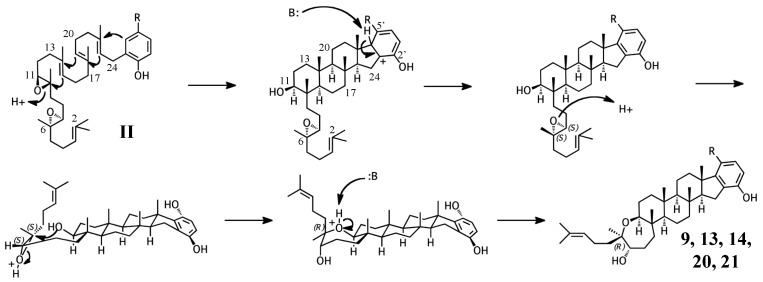
Putative cyclization routes of group II sponge merotriterpenoids, derived from 6,7-10,11-diepoxyhexaprenyl diphosphate, including: **9**, **13**, **14**, and haliclotriols A (**20**) and B (**21**).

Adociasulfates and related meroterpenoids of group II are likely derived from a diepoxy precursor (Figure 5). Three of five members of this group exhibit a 5′ glycolic acid substitution akin to **10**, **15**, and **16** [6,12,27]. The first cyclization event of **9** may mirror that of **2** from group I, with the opening the 10,11-epoxide and establishment of the adociasulfate core. The second cyclization would then involve the opening of the 6,7-epoxide by back-side attack of the C11 hydroxyl at the more-substituted C6 position in a typical acid-catalyzed epoxide opening. This would result in the formation of a seven-membered ring and an inversion of C6 stereochemistry. Assuming a *pro*-chair conformation would position C6 into a pro-(*R*) configuration relative to the C11 hydroxyl attack, resulting in the axial-oriented terminal olefin. For group I compounds, the lack of the 6,7-epoxide likely allows for inclusion of the 2,3-terminal alkene in the second cyclization event (Figure 4A), whereas all group II compounds display a free terminal olefin. This may reflect an enzymatic preference for protonation of epoxides over alkenes, resulting in early termination of the cyclization cascade.

### 2.3. Group III compounds

**Figure 6:**
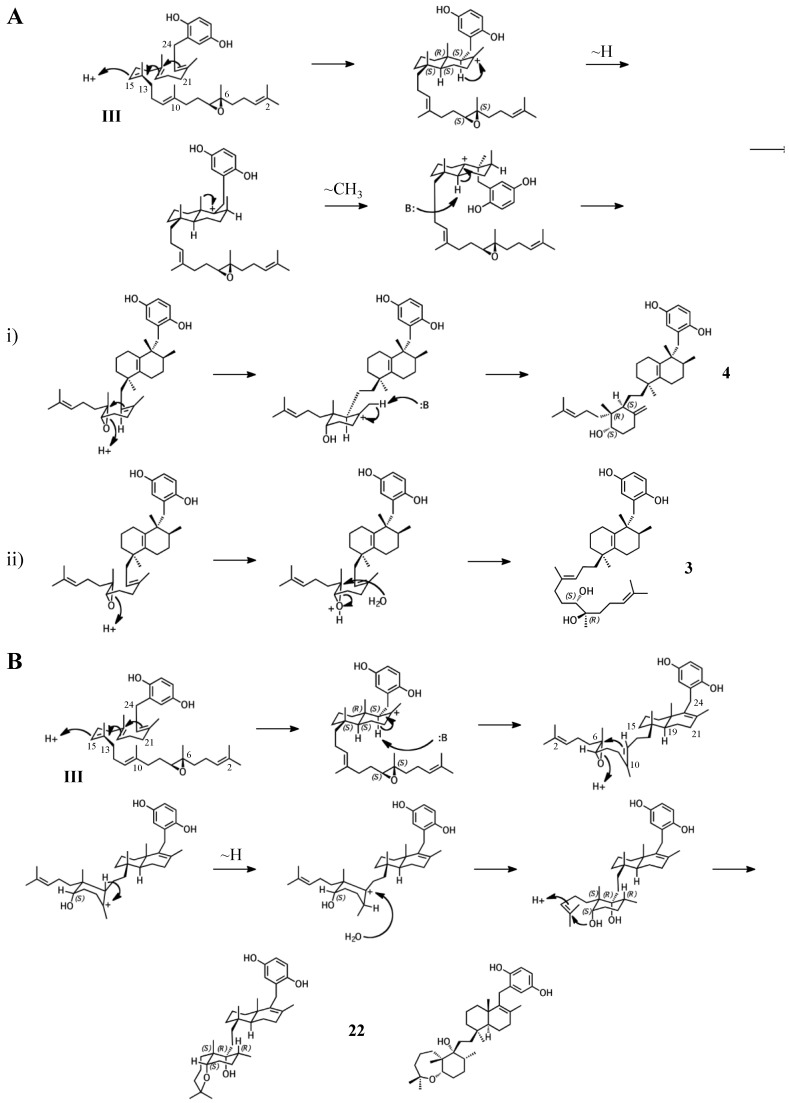
Putative cyclization routes of group III sponge merotriterpenoids, derived from 6,7-epoxyhexaprenyl diphosphate, including: (**A**) **3**, **4**, (**B**) and shaagrockol C (**22**).

Group III merotriterpenoids are likely derived from a 6,7-epoxy precursor. This group is characterized by a lack of fusion to the aromatic ring and quenching by water. In the proposed cyclization of **3** and **4**, initiation by protonation would result in a bicyclic drimane-like skeleton that undergoes rearrangement before deprotonation by an active-site base, yielding a highly stable tetra-substituted double bond and unique configurations of methylated carbons (Figure 6A) [9]. The first cyclization event of **3** and **4**, involving the 14,15-, 18,19-, and 22,23-olefins, likely involves prearrangement of the substrate in a chair-chair orientation, placing the remaining linear terpene chain in a pre-equatorial position. For **4**, protonation of the 6,7-epoxide would initiate the second cyclization event involving the 10,11-olefin (Figure 6(Ai)), while hydrolysis of the epoxide would lead to **3** (Figure 6(Aii)). The first cyclization event of shaagrockol C (**22**) would also produce a bicyclic system, though deprotonation would occur prior to any rearrangement, yielding a tetra-substituted alkene (Figure 6B) [28]. The second cyclization would be similar to that of **4.** Prearrangement of the remaining linear portion of the substrate in a boat conformation, followed by hydride transfer from the C11 axial hydrogen to the C10 carbocation would allow for the (*R*) configuration at C10, as opposed to the (*S*) configuration that would result from a chair prearrangement and analogous hydride shift. Water would attack the C11 carbocation with inversion of stereochemistry. The net result of this dramatically different cyclization route is that the newly formed ring of **22** would incorporate an axial hydroxyl group in place of a proton at C11. Thus, **22** and **4** display the same relative configuration about C11, despite differing absolute configurations. Finally, the C7 hydroxyl would initiate a final cyclization with the 2,3-alkene, forming a 7-membered terminal heterocycle. Shaagrockol B, isolated together with **22**, is the oxidation product of **22** about the 22,23-alkene and is likely not enzymatic in origin [28].

### 2.4. Group IV compounds

**Figure 7:**
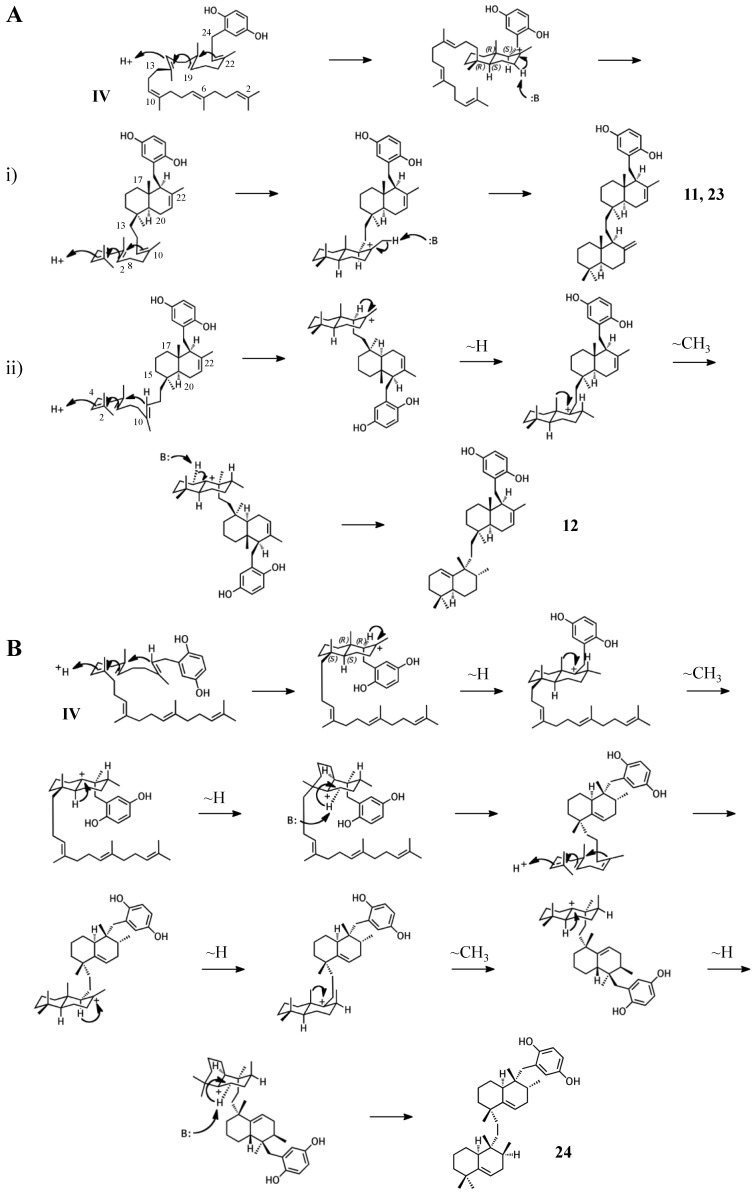

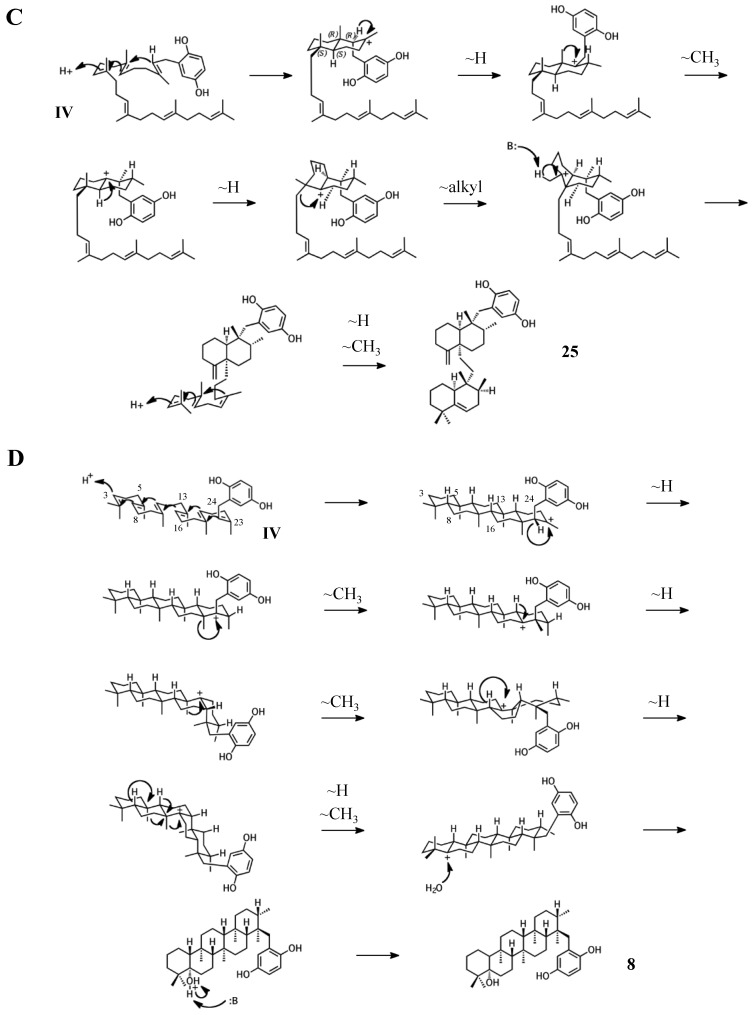
Putative cyclization routes of group IV sponge merotriterpenoids, derived from hexaprenyl diphosphate, including: (**A**) **11**, **12**, adociaquinol (**23**), (**B**) toxiusol (**24**), (**C**) akaterpin (**25**), and (**D**) **8**.

The remaining six known sponge merotriterpenoids of group IV are likely derived from a substrate lacking epoxidation. The majority of these compounds undergo complex cyclizations followed by rearrangements, as evidenced by their atypical methyl positions. Like the group III compounds, none of the group IV members exhibit fused rings with the aromatic moiety, suggesting that aromatic ring fusion requires the presence of the 10,11-epoxide. Another common feature between groups III and IV is the absence of 5′ glycolic acid substitution. For **11**, **12**, and adociaquinol (**23**), the proposed initial cyclization would, like the group III compounds, yield a two-ring system, but would differ from these in the prearrangement of the substrate in a boat-chair conformation, placing the linear terpene chain in the less favorable axial position. (Figure 7A) [29]. Due to the absence of the 6,7-epoxide, the second cyclization event of **11**, **12**, and **23** would include the terminal olefin that was excluded by the group III compounds. The second cyclization event of **11** and **23** likely resembles the initial cyclization event of **3** and **4**, involving preorganization of the substrate in the chair-chair orientation that places the terpene chain in the more stable equatorial position (Figure 7(Ai)). Deprotonation at the C10 methyl would introduce the exocyclic alkene. The second cyclization event of **12** would involve a chair-boat conformation, placing the ring system established in the first cyclization event in the axial position, with deprotonation at C5 following both a hydride and methyl shift to form the trisubstituted alkene (Figure 7(Aii)). Cyclization of the initial bicyclic ring system of toxiusol (**24**) likely involves the chair-boat conformation, placing the hydroquinone in the axial position (Figure 7B) [7,29]. Two hydride shifts and a methyl transfer would occur prior to deprotonation to complete first cyclization. The second cyclization event of **23** would occur via the chair-chair conformation similar to **11** and **23**, but a series of hydride transfers would place the trisubstituted alkene on the opposite ring relative to **12**. The cyclization of akaterpin (**25**) likely follows a similar cyclization scheme as **24** but would involve an alkyl shift during the first event, relocating the remaining linear isoprene chain from C14 to the bridgehead carbon, C19 (Figure 7C) [4]. The final sponge merotriterpenoid, **8**, can be reached with a single proton-initiated cascade followed by extensive rearrangement. The substrate is likely prearranged in the antipodal conformation, the opposite orientation of the group I and II cyclizations, such that the end result appears structurally distinct from the sterol-like adociasulfate core of group I and II meroterpenoids (Figure 7D) [10]. In total, five hydride shifts and four methyl shifts would need to occur before an attack by water at the bridgehead carbon C7.

From this model of the origin of adociasulfates, it should be clear that all sponge merotriterpenoids of the hydrobenzoquinone family are related biosynthetically. In each adociasulfate discovery reported, mixtures of compounds from multiple groups were identified, suggesting a common synthetic route that is independent of the epoxidation state of the substrate [7,8,9,12,27,29]. Of this class of compounds, all but one member has been isolated from sponges within the family *Chalinidae*. The exception is **25**, which was reportedly discovered from *Callyspongia sp* [4]. Though *Callyspongia* is a member of the same order as *Chalinidae* (order *Haplosclerida*), *Callyspongia* is far enough removed in this case to be considered unrelated (Mary Kay Harper, personal communication, 2016). Thus, these compounds can be used as taxonomic identifiers, potentially due to a shared biosynthetic pathway.

## 3. Considerations of the enzymatic origin of sponge merotriterpenoids

Only a few key biosynthetic steps are required for all four groups of merotriterpenoids described above: aromatic prenylation, proton-initiated cyclization, and sulfation. Epoxidation also occurs for the majority of these compounds, with the exception of group IV. The potential enzyme families responsible for these key steps of adociasulfate construction are discussed in this section. The source of the terpene and benzoquinone precursors is also considered, as these metabolites can be derived from multiple routes and the enzymes involved in their synthesis may be components of an adociasulfate biosynthetic gene cluster. In addition to the enzymatic origins of sponge merotriterpenoids, the identity of the producing organism is taken into account, as this will dramatically affect the genetic organization of the pathway.

### 3.1. Origin of precursors

The majority of the adociasulfate structure is constructed of five-carbon isoprene units. There are two known biosynthetic pathways for isoprene production: the mevalonate (MEV) pathway, which provides the precursors for steroids in eukaryotes but is also present in some bacteria, and the 1-deoxy-D-xylulose-5 phosphate (MEP/non-mevalonate) pathway unique to plants, bacteria, and some parasites. Both of these are considered primary metabolic pathways. It is possible that the adociasulfate pathway draws IPP directly from an endogenous metabolite pool and lacks any dedicated genes for IPP/DMAPP synthesis. However, the producing organism’s native isoprene source does not necessarily imply that pathway’s involvement in secondary metabolism. Bacteria normally lacking the MEV pathway are known to incorporate horizontally acquired MEV pathway genes into meroterpenoid biosynthetic clusters as a pathway-specific source of IPP/DMAPP [30,31,32,33,34,35,36,37,38,39]. Some MEP pathway bacteria contain duplications of MEP genes in secondary metabolite clusters [40,41]. The role of these seemingly redundant genes may be to enhance production of precursor metabolites or to establish regulation of early steps in the pathway. Thus, copies of MEV or MEP pathway genes might be involved in meroterpenoid production. However, as there is no evidence to suggest one isoprene pathway being involved over the other, adociasulfate pathway identification should focus on the biosynthetic steps unique to merotriterpenoids. The presence of isoprene pathway elements should be considered a secondary indication of a terpene pathway.

The adociasulfate prenyl donor, consisting of six isoprene units, is almost certainly a product of a *trans* isoprenyl diphosphate synthase. Isoprenyl diphosphate synthases are soluble, Mg^2+^-dependent prenyltransferases (PTases) mechanistically related to aromatic UbiA-like PTases [42,43]. These enzymes are responsible for producing prenyl diphosphates of different lengths for various biological functions, including polyprenyl diphosphates of 30–50 carbons used in ubiquinone and menaquinone biosynthesis, and the FPP used to make squalene in steroid biosynthesis. Isoprenyl diphosphate synthases are sometimes components of meroterpenoid gene clusters [30,31,32,33,34,35,38,40]. Their inclusion in secondary metabolite pathways may reflect a selection mechanism for a particular length polyprenyl substrate, establishing a distinct substrate pool for meroterpenoid biosynthesis separate from the endogenous IPP pool. However, native isoprenyl diphosphate synthases are likely capable of providing the prenyl substrate for secondary metabolism. 

**Figure 8:**
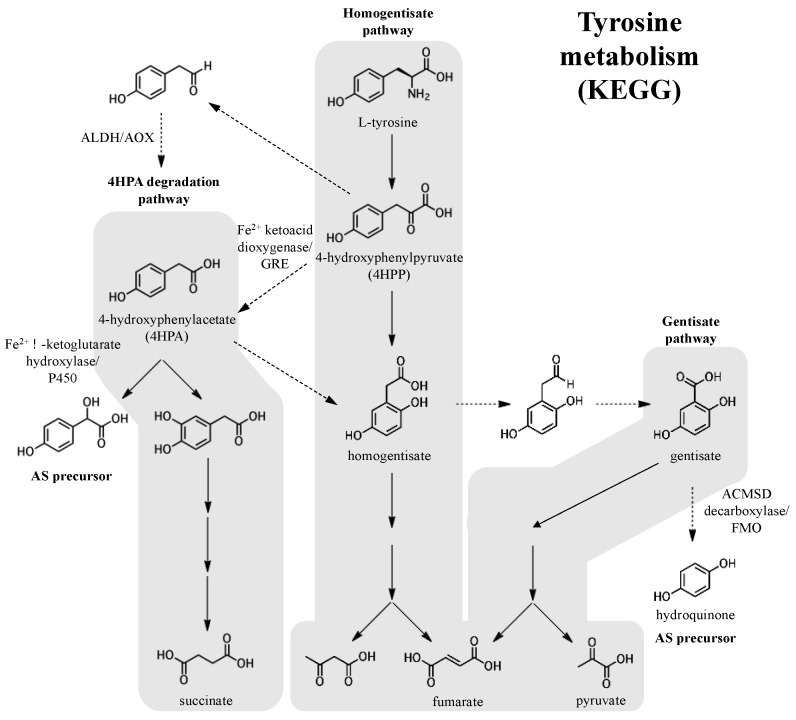
Potential biosynthetic origins of the aromatic adociasulfate prenyl acceptor from tyrosine metabolism pathways, as mapped by the Kyoto Encyclopedia of Genes and Genomes (KEGG). Dashed arrows indicate possible or uncharacterized enzymatic transformations.

Like prenyl diphosphates, quinones can also derived from primary metabolic pathways like the phenylalanine/tyrosine pathway, from which hydroquinone and 4-hydroxyphenylacetate (4HPA), a potential pre-hydroxylation precursor of the 5′-glycolic acid substituted adociasulfates, can be derived (Figure 8). 4HPA may be derived from 4-hydroxyphenylpyruvate (4HPP), a product of tyrosine degradation. Oxidative decarboxylation, such as that catalyzed by 4-hydroxyphenylpyruvate (4HPP) dioxygenase, an Fe^2+^-dependent internal ketoacid dioxygenase, could be used to generate 4HPA from 4HPP [44]. Alternatively, 4HPA could potentially be obtained from 4HPP via 4HPA decarboxylase, such as the enzyme of *Clostridium difficile* that produces *p*-cresol from 4HPA and is a member of the glycyl radical enzymes (GRE) of the radical-SAM superfamily [45]. In a less direct route, decarboxylation of 4HPP to the aldehyde with subsequent oxidation to 4HPA by either an aldehyde dehydrogenase (ALDH) or an aldehyde oxidase (AOX) could be possible [46,47]. Both the NAD(P)^+^-dependent ALDHs and flavin-dependent molybdenum/tungsten AOXs are described as broad-substrate and are largely uncharacterized. Subsequent hydroxylation of the 4HPA acyl side-chain could be carried out by an α-ketoglutarate-dependent Fe^2+^ enzyme or a cytochrome P450 (P450) [48,49]. 4HPA could also enter into the homogentisate pathway, where hydroquinone could be obtained from homogentisate in a few enzymatic steps [50,51]. Hydroquinone could be derived from gentisate by decarboxylation, potentially requiring a nonoxidative decarboxylase like 5-carboxyvanillate or γ-resorcylate decarboxylase, both members of the ACMSD decarboxylase family [52,53,54]. Oxidative decarboxylation of aromatic substrates can also be carried out by flavin monooxygenases (FMOs) [55]. Though it is unclear whether tyrosine metabolism factors into meroterpenoid biosynthesis, enzymes similar to these are capable of supplying the prenyl acceptor.

The majority of meroterpenoid pathways contain genes responsible for providing or modifying existing aromatic precursors, but these genes represent a variety of distinct biosynthetic routes. Hydroquinone prenyl acceptors of known meroterpenoid pathways are derived primarily from polyketides [31,32,34,38,56,57,58,59,60], but can also be derived from tyrosine [41,61], and from the carbohydrate sedoheptulose 7-phosphate [33]. Another possibility is that the prenyl acceptor is extensively modified after the initial prenylation event, as is the case in ubiquinone synthesis. 4-hydroxybenzoate (4HB) and homogentisate, similar in structure to 4HPA and hydroquinone, are known prenyl acceptors in the ubiquinone and plastoquinone/tocopherol pathways, respectively [62,63]. Prenyl-4HB/homogentisate could be decarboxylated and then hydroxylated to generate the precursor of adociasulfate cyclization. From the examples described here, merotriterpenoids are likely to include specific genes devoted to hydroquinone synthesis.

### 3.2. Prenylation

Prenyltransferase is the first true step in adociasulfate biosynthesis. A variety of aromatic prenyltransferases (PTases) are known to generate products resembling the linear adociasulfate precursors shown in Figure 2. The earliest to be characterized of these enzymes is 4HB-PTase, which is involved in ubiquinone biosynthesis [62,64]. 4HB-PTases are present in all forms of life, as ubiquinone is an essential component of biological redox reactions like the electron transport chain. The mechanism of prenyl transfer by UbiA, the 4HB-PTase of *E. coli*, involves activation of the isoprene diphosphate to form a carbocation, initiating the electrophilic addition to 4HB in a Friedel-Crafts type alkylation [65,66]. UbiA and related PTases are broadly substrate selective in vitro, especially with regard to the length of isoprenes that can be incorporated into their product [62,67,68]. UbiA also exhibits broad substrate specificity for prenyl acceptors, provided that these substrates are *para*-alcohol- or amino-substituted benzoates [69]. In fact, membrane-associated aromatic PTases utilize a wide variety of aromatic prenyl acceptors in the biosynthesis of plastoquinones/tocopherols, menaquinone, and even secondary metabolites; a testament to their vast biosynthetic potential [70]. It is likely, owing in particular to their accommodation of variable isoprene chain lengths, that membrane aromatic PTases are involved in sponge meroterpenoid biosynthesis.

Prenylation is not unique to the UbiA-like PTases, however, and could be accomplished by other enzyme families. The ABBA-family of aromatic PTases, so named for their alternating, antiparallel α -β-β-α folds (dubbed the PT-fold or PT-barrel), are soluble aromatic prenyltransferases involved in secondary metabolism of bacterial and fungal natural products [71,72]. Though ABBA PTases are broadly selective with regard to the aromatic prenyl acceptor, they are restricted in the length of the prenyl donor to two or fewer isoprene units. Only one ABBA PTase is known to accept FPP as a prenyl donor [30]. Despite the significant role of ABBA PTases in secondary metabolism, the comparison between PTase families better supports the idea that a membrane-associated PTase is involved in sponge meroterpenoid biosynthesis.

### 3.3. Cyclization

Cyclization of triterpenes is an electrophilic reaction catalyzed by class II terpene cyclases. Class II triterpene cyclases of the bacterial squalene-hopane cyclase (SHC) and eukaryotic oxidosqualene-lanosterol cyclase (OSC) families are known for both their broad substrate selectivity and their extreme product diversity in vitro [73,74,75,76]. This product diversity is related to the proton-initiated mechanism of cyclization. Carbocation-mediated rearrangements occur frequently, and similar substrates may be cyclized differently depending on where they are protonated, which depends on both substrate and enzyme and shape. Despite this, cyclization is a highly stereospecific reaction, resulting in characteristic configurations about the chiral bridgehead and methyl-substituted carbons. The fit of the substrate within the cyclase active site likely plays a large role in determining the arrangement of the rings in the final product. Many adociasulfates display sterol-like stereochemistry within rings A-C, indicative of the “prechair” conformation assumed by group I and II adociasulfates prior to cyclization that is characteristic of both sterol and hopene cyclizations (Figure 4) [73]. Group III and IV adociasulfates exhibit bicyclic skeletons, which are also products of SHC/OSCs in vitro [73,76]. As sponge merotriterpenoids display features characteristic of proton-initiated cyclization, including complex rearrangements and substrate-dependent patterns of carbon skeletons (Figure 2), an SHC- or OSC-like cyclase is likely involved in their synthesis.

Class II terpene cyclases do not utilize linear meroterpenoid substrates in nature, but are nonetheless capable of performing the chemistry required of a putative adociasulfate cyclase. Both SHCs and OSCs can cyclize linear hydroquinone meroterpenoids in vitro [75,77,78,79]. In these examples, SHCs are able to cyclize the prenyl side chain of the linear meroterpenoid substrate, but their products lack fusion of the aromatic moiety to the terpene ring system [77,79]. The OSC lupeol synthase (LUP1) from *Arabidopsis thaliana*, however, is capable of fusing the aromatic indole ring of its epoxide substrate to the prenyl side chain [78]. This is similar to the epoxide-dependent aromatic ring fusion observed for group I and II adociasulfates (Figure 4 and Figure 5). This would suggest that the presence and position of epoxides determine which type of skeleton will be formed. In this way, a single class II terpene cyclase could be responsible for the production of all sponge merotriterpenoids. In an example of substrate-dependence on cyclization, tetraprenyl-β-curcumene cyclase of *Bacillus subtilis* is capable of utilizing both a linear, monocyclic C_35_ terpenoid substrate to generate a fused four-ring skeleton strongly resembling group I and II adociasulfates, and squalene to produce a fused bicyclic drimane-like skeleton similar to group III and most group IV adociasulfates [80]. In this case, the structural differences between the linear, head-to-tail tetraprenyl-β-curcumene and the tail-to-tail squalene direct the outcome of the cyclization event. Adociasulfate cyclizations sometimes involve heterocycle formation, presumably involving hydroxyls produced by epoxide ring openings. SHCs are capable of heterocycle formation in this way [81]. In general, SHCs exhibit greater substrate flexibility than OSCs and can accept a variety of terpene substrates in vitro, including 2,3-oxidosqualene [73,82]. Thus, it is likely that a bacterial SHC-like enzyme is responsible for adociasulfate cyclization. 

Though one could envision the adociasulfate biosynthetic pathway containing an SHC-like terpene cyclase, natural product pathways often include atypical enzymes capable of performing similar chemistry rather than the more recognizable class II terpene cyclases. For example, several fungal indole meroterpenoid pathways utilize a novel family of small, membrane-bound meroterpenoid cyclases (MTCs) capable of proton-initiated cyclization [83]. One of these enzymes, PaxB, has been shown to cyclize doubly epoxidized substrates similar to those predicted for group II sponge merotriterpenoids [84]. The resulting compound, paspaline, is remarkably similar to adociasulfates in that it, too, contains a heterocycle formed after an initial epoxide opening cyclization event, using the resulting hydroxyl group in the second cyclization reaction. MTCs have thus far only been reported to cyclize merosesquiterpenoid and meroditerpenoids, but it appears plausible that such enzymes could catalyze longer cyclizations, such as that predicted for **8** (Figure 7D).

### 3.4. Epoxidation

Epoxidation of squalene in eukaryotes is carried out by squalene monooxygenase (SM), a membrane-bound flavin-dependent protein that requires molecular oxygen and reduced NADPH, as well as a P450 reductase partner [85]. The requirement for a P450 reductase is unique to SM amongst FMOs, as there is no structural relationship between SM and P450s, but several groups of FMOs are known to require other flavin reductase partners [55]. There is evidence that a second, non-P450 type flavin reductase may be also be able to supply reduced NADPH to SM [86]. There is also a precedent for SM in secondary metabolism. The diterpene phenalinolactone, produced by a *Streptomyces* strain, includes an SM homolog in its biosynthetic gene cluster [87]. This SM homolog is believed to introduce an epoxide at the terminal olefin of the C20 geranylgeranyl diphosphate substrate. SM produces a single isomer of oxidosqualene, introducing an oxirane ring at the terminal 2,3-alkene in the (*S*) configuration. However, due to the rigid specificity of SM for terminal olefins it is more likely that an unrelated monooxygenase is involved in sponge merotriterpenoid epoxidation. For example, non-SM FMOs related to oxidative genes of the ubiquinone pathway have been identified in fungal indole terpenoid gene clusters, such as that of xiamycin [2,88]. Additionally, P450 monooxygenases are involved in oxidative tailoring reactions in numerous natural product pathways and are capable of performing a wide variety of chemical modifications on diverse substrates, including epoxidation. All P450s obtain reduced flavin via a P450 reductase partner, similar to SM [89]. Owing to their incredible diversity in both function and substrate specificity, either FMOs or P450s are a more likely candidate for epoxidation than SM in the adociasulfate pathway. 

### 3.5. Sulfation

The final step in the synthesis of adociasulfates is sulfation of the hydroquinone moiety. In eukaryotes, sulfation is carried out by sulfotransferases (SULTs) that utilize 3′-phosphoadenosine 5′-phosphosulfate (PAPS) as a sulfonate (SO_3_^−^) donor. Though SULTs are less prevalent in bacteria than in eukaryotes, sulfation has been incorporated into secondary metabolism. SULT domains have been identified within polyketide synthases to generate sulfated products, or, in one case, sulfation activates a substrate for decarboxylation [90]. The role of sulfation in adociasulfate activity can only be guessed, as the native biological function of adociasulfates is not known. However, with regard to kinesin, the sulfates only prevent membrane penetration and do not affect inhibition [12,91]. Sulfation could be a mechanism for elimination from the sponge to avoid toxicity associated with kinesin inhibition, or it could enhance secretion to facilitate exposure to predators. Not all sponge merotriterpenoids are sulfated, however, but these compounds have not been tested for kinesin inhibition [5,6]. It has been suggested that an analog of **14** containing an esterified glycolic acid moiety and lacking sulfation might be membrane permeable and still inhibit kinesin, making it a good anticancer lead [12]. **21** and haliclotriol triacetate closely resemble this hypothetical analog and should be screened for kinesin inhibition [6]. Nonetheless, sulfation is not essential for adociasulfate biosynthesis, and the genes involved need not reside in the same gene cluster or even the same genome as the rest of the pathway. While a microbial symbiont may produce adociasulfates, the host could be responsible for their sulfation.

## 4. Concluding remarks

To date, adociasulfates remain the only known natural product kinesin inhibitors that compete with MTs for binding. Until recently adociasulfates were thought to form MT-mimicking aggregates, bringing into question their potential as drugs or mechanistic probes [92]. It is now understood that adociasulfates bind kinesin in a 1:1 interaction [12]. In light of these findings, it is crucial to point out the unlikeliness of kinesin inhibitors RBL and NSC 622124 to behave as expected in biochemical or cell-based investigations. Adociasulfates are the only experimentally validated inhibitors to compete with MTs for binding kinesin at a single-molecule level. Thus, there exists some urgency to achieve sustainable adociasulfate production.

**Figure 9:**
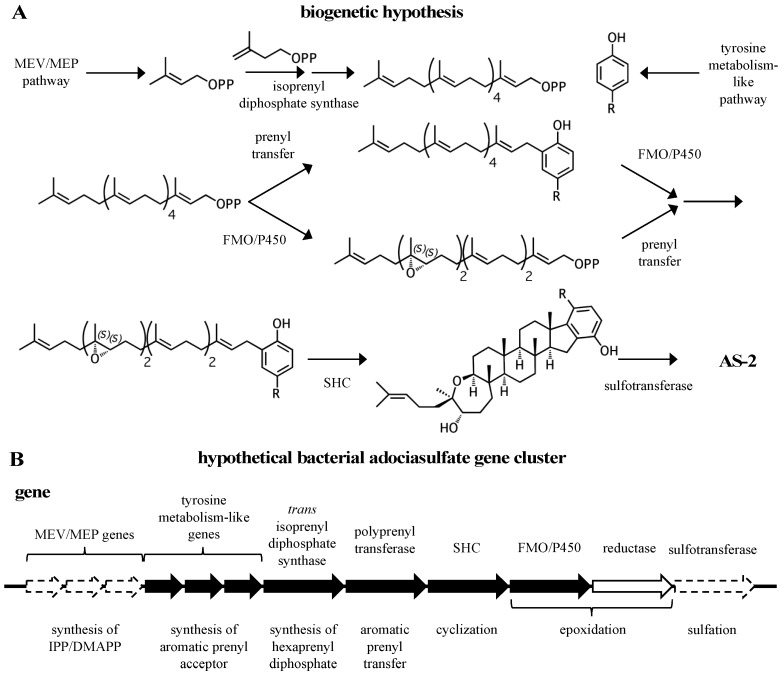
A biogenetic hypothesis for the adociasulfates. (**A**) A biosynthetic scheme summarizing the proposed biogenetic hypothesis for the origin of adociasulfates. (**B**) A hypothetical adociasulfate gene cluster was constructed based on the most probable biosynthetic origin, as addressed in this review. In this scenario, the pathway is assumed to be part of a bacterial genome. Black genes represent those directly involved in biosynthesis, white genes are those indirectly involved in biosynthesis, and those bordered with a dashed line have the potential to be entirely absent from the cluster.

A general biogenetic hypothesis can be made based on the proposed structural relationships between hydroquinone merotriterpenoids (Figure 9A). Proton-initiated cyclization from variable substrates, including non-epoxides, suggests a squalene-hopene cyclase. The positions of the epoxides in the linear precursors suggest that an FMO or P450 may be responsible. The head-to-tail linear triterpene precursor supports the idea that a polyprenyl synthase supplies the precursor of cyclization. These observations, which encompass the more distinct features of the adociasulfate structure, imply a bacterial origin (Figure 9B). This biogenetic hypothesis is supported by the recent discovery of a meroterpenoid pathway from marine cyanobacteria, which are often involved in symbioses with sponges and other invertebrates [93]. The merosterols are meroditerpenoids that greatly resemble adociasulfates. Their biosynthetic pathway incorporates elements of the MEP pathway for isoprene production, and both carbohydrate and tyrosine metabolism for synthesis of the PHB moiety. An UbiA-like PTase and SHC-like cyclase were shown to generate a cyclized meroterpenoid product, and several genes encoding oxidative proteins are present, including two P450s, presumably to introduce modifications to the aromatic ring. Despite these similarities, no biosynthetic pathways for sponge meroterpenoids have ever been identified for comparison. In only one case has a producing organism been claimed to have been identified—for the production of avarol, a merosesquiterpenoid, by the sponge *Dysidea avara*. In these studies, avarol was traced to a specific sponge cell type and production was later observed from an axenic primary sponge culture [94,95]. However, no publications have followed these studies in nearly 18 years. Thus, while the possibility exists that adociasulfates and related meroterpenoids are sponge-derived, or that merosesquiterpenoid biosynthesis may differ substantially with that of merotriterpenoids, the biosynthetic origin of sponge merotriterpenoids that is most consistent with their structure is bacterial.

Though the structure of adociasulfates favors symbiont- over host-derived production, no clear verdict can be reached without experimental investigation. Clues as to what types of enzymes are responsible have been described here. Targeted searches of genes with these functions could help to identify the adociasulfate pathway. Metagenomic approaches may complicate data interpretation in that several to hundreds of gene homologs may be identified within a single metagenome, especially for those genes related to primary pathways, such as *ubiA*. A comparative metagenomics approach may resolve these issues, in which the metagenomes of nonproducing *Chalinidae* sponges are sequenced alongside adociasulfate-producing specimens. Care must be taken to collect and prepare separate samples for analysis by chemical and DNA sequencing approaches. Following the guidelines for pathway identification laid out in this review may result in successful recognition of a meroterpenoid pathway, paving the way for biosynthetic approaches to solve the supply problem that surrounds these valuable compounds. More importantly, a thorough analysis of compound structure can reveal valuable information regarding the compound’s origin. This strategy can be used as a general approach in the discovery of natural product biosynthetic pathways.
